# Heterogeneity of anoikis in triple-negative breast cancer subtyping and therapeutic implications

**DOI:** 10.1016/j.gendis.2024.101255

**Published:** 2024-03-04

**Authors:** Kun Fang, Suxiao Jiang, Zhengjie Xu, Meng Luo, Changsheng Yan

**Affiliations:** a Department of Surgery, Yinchuan Maternal and Child Health Hospital, Yinchuan, Ningxia 750001, China; b Department of Surgery, The First Affiliated Hospital of Harbin Medical University, Harbin, Heilongjiang 150081, China

Triple-negative breast cancer (TNBC) is an inherently heterogeneous malignancy at the biological and clinical levels, occupying around 15% of diagnosed breast cancer cases and being the most lethal breast cancer subtype to combat.^1^ Anoikis is a programmed cell death that occurs when cells detach from the correct extracellular matrix, thereby destroying integrin connections.^2^ However, the role of anoikis expression pattern and prognosis in TNBC is still unrevealed. Here, TNBC presented three heterogeneous phenotypes in anoikis, and each subtype owned unique molecular, and clinical characteristics as well as diverse responses to chemotherapy, targeted therapies, and immune checkpoint blockade. The anoikis-based scoring system can accurately estimate patient survival, which was remarkably superior to well-established clinical parameters. Patients with a low anoikis score were predicted to well respond to immune checkpoint blockade, and compounds were identified to be appropriate for those with a high anoikis score. The genes from the anoikis-based scoring system were aberrantly expressed in bulk TNBC versus normal tissues, and specifically expressed in TNBC single cells. Each of them was linked with a TNBC prognosis. Altogether, our study sheds light on the anoikis landscape of TNBC, which will contribute to further understanding of anoikis and formulating an appropriate treatment choice to defeat TNBC.

To clarify this, 434 anoikis genes were queried from the GeneCard database (Table S1). Transcriptome data on 116 TNBC and 113 normal tissues were obtained from The Cancer Genome Atlas (TCGA) (Table S2). The GSE58812 (*n* = 107) from the Gene Expression Omnibus,^3^ and METABRIC (*n* = 298) datasets were utilized for external verification. Among 434 anoikis genes, 101 presented significant down-regulation, and 94 presented significant up-regulation in 116 TNBC versus 113 normal specimens (Fig. S1A, B), suggesting abnormal anoikis in TNBC. These differentially expressed genes were strongly associated with tumorigenic pathways, such as pathways in cancer, PI3K-Akt/Rap1/p53/HIF-1/FoxO signaling pathways, and apoptosis (Fig. S1C), highlighting their importance in TNBC. We further conducted univariate Cox regression analysis on the anoikis genes and identified 15 prognostic genes (Fig. S1D). Based on the expression of these prognostic genes, the 116 TNBC samples were categorized into three subtypes named C1, C2, and C3, which were clearly distinguished by consensus matrix and cumulative distribution function results (Fig. 1A; Figs. S2A–C). The principal component analysis confirmed the significant differences among the three anoikis subtypes (Fig. 1B). Prognostic outcomes also varied among the subtypes, with C2 showing the worst prognosis, followed by C3 and C1 (Fig. 1C). The expression of the prognostic genes was also diverse among the subtypes (Fig. S1E). Figure S1F illustrates the heterogeneity in clinical features across the subtypes. In addition, most immune cells were lowly abundant in the C1, but were highly abundant in the C2 and C3 subtypes (Fig. 1D). Immune checkpoint molecules CD200, CD276, CD80, CD86, HAVCR2, LAIR1, NRP1, and TNFRSF14/25/4/8 presented the highest expression in the C2, followed by the C3 and C1 subtypes (Fig. S3A). This indicated that the C2 owned the highest possibility to respond to immune checkpoint blockade. As illustrated in Figure 1E, most hallmarks displayed the lowest activity in the C1, with the highest activity in the C2 and C3 subtypes, partly explaining why the C1 owned the best prognosis. Next, the IC50 values of chemotherapy or targeted drugs were estimated in each subtype. The C1 exhibited the highest responses to cisplatin, gefitinib, and gemcitabine, with the highest responses to docetaxel and sunitinib for the C2, without the difference in sorafenib response among the subtypes (Fig. S3B).

**Figure 1:**
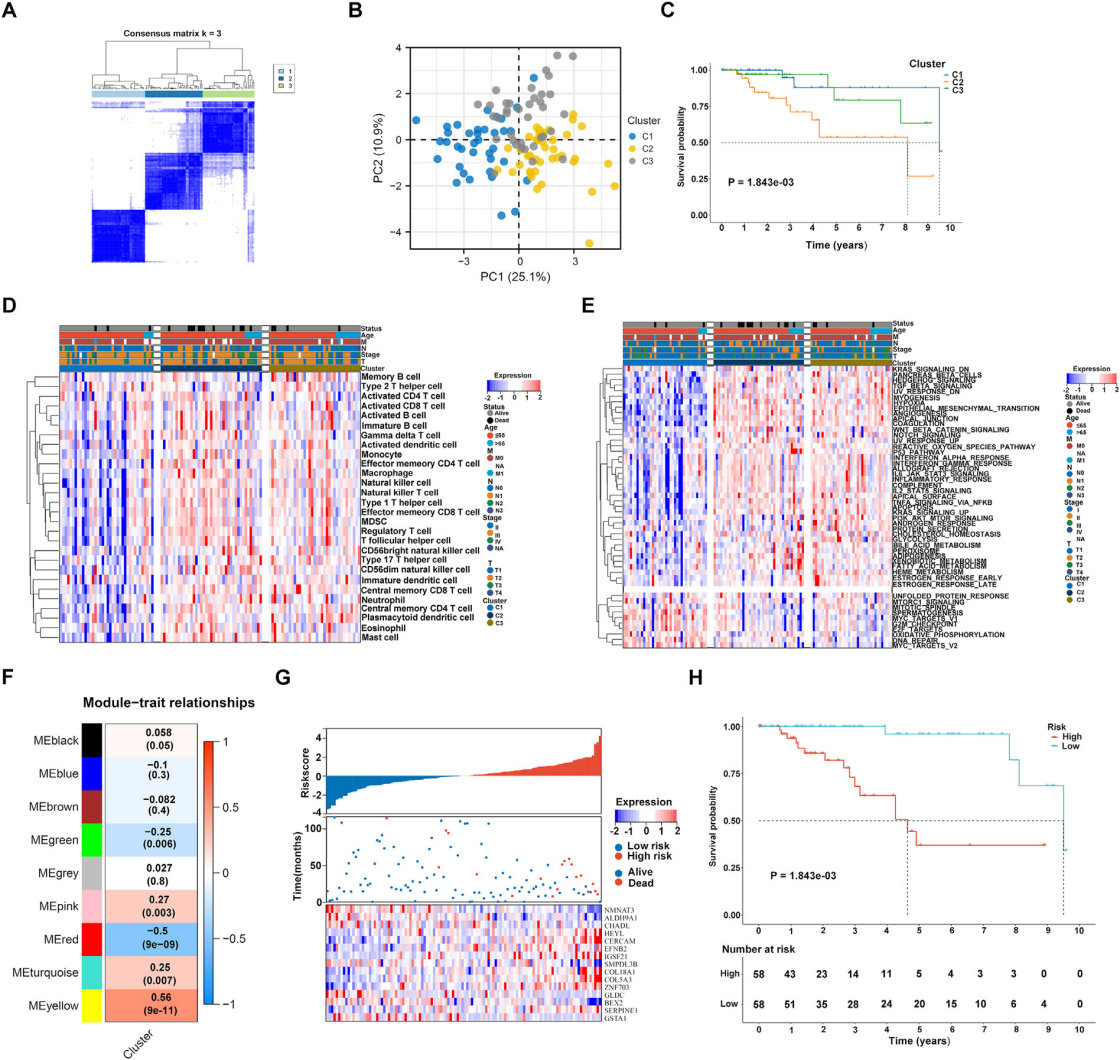
Construction of three anoikis-based subtypes and risk model. **(A)** Consensus matrix heatmap at k = 3 in accordance with the expression of the prognostic differentially expressed anoikis genes. **(B)** The principal component analysis plots demonstrating the distinction of the three anoikis-based subtypes. Each subtype was marked by a unique color. **(C)** Survival probability of the three anoikis-based subtypes. **(D)** The abundance of different immune cells across the three subtypes. Blue to red denotes infiltration from low to high. **(E)** The activity of well-established hallmarks across the subtypes. **(F)** Heatmap of the relationships of the co-expression modules with anoikis subtyping. **(G)** Distribution of anoikis score, survival time, and expression of the identified genes in TCGA TNBC samples. **(H)** Survival probability of TCGA TNBC patients with a low or high anoikis score.

We then conducted a weighted gene co-expression network analysis to identify genes related to anoikis subtyping (Fig. S4A). After determining an appropriate soft threshold power of 12 to construct a scale-free network (Fig. S4B), we merged genes with similar expression patterns into nine co-expression modules (Fig. S4C, D). The yellow module showed the strongest association with anoikis subtyping (*r* = 0.56, *P* = 9e-11) (Fig. 1F), with module membership strongly related to gene significance (*r* = 0.8, *P* = 1.5e-120) (Fig. S4E). We selected 536 genes from the yellow module as anoikis subtyping-related genes (Table S3), which were significantly associated with cancer pathways, PI3K-Akt/TGF-beta signaling pathways, ECM-receptor interaction, *etc* (Figs. S4F–I), highlighting their roles in TNBC. Among these genes, 52 showed significant associations with TNBC prognosis (Table S4). We then performed LASSO analysis to define an anoikis scoring system using the formula (Fig. S5A, B). Based on the median score, samples were divided into low or high anoikis score subgroups (Fig. 1G). Survival analysis showed a significant difference between subgroups. Patients with a high anoikis score had a notably poorer prognosis compared with those with a low anoikis score (Fig. 1H). The area under curve values for one, three, and five-year endpoints were 0.841, 0.958, and 0.958, respectively, indicating that the anoikis scoring system accurately predicted patient survival (Fig. S5C). This survival difference between subgroups was proven in the GSE58812 and METABRIC datasets as well (Figs. S5D–G). Furthermore, there was a significant association between the anoikis score, T, N, M, stage, and TNBC patient survival (Fig. S6A). The anoikis score was identified as an independent risk factor for prognostic outcomes (Fig. S6B). Time-independent receiver operating characteristic curves demonstrated the superior predictive ability of the anoikis score compared with clinical parameters (Fig. S6C). A nomogram was then developed based on the anoikis score, T, N, M, and stage to estimate prognosis (Fig. S6D). Calibration curves confirmed the nomogram's strong predictive accuracy (Fig. S6E). Additionally, the tumor mutation burden survival curve revealed that patients with a high tumor mutation burden had a significantly longer survival time, and those with both a high tumor mutation burden and low risk had the most favorable prognosis (Fig. S6F, G).

Immune cells including central memory CD4 and CD8 T cells, regulatory T cells, CD56bright and CD56dim natural killer cells, macrophages, monocytes, natural killer T cells, and neutrophils presented the remarkably stronger abundance in high versus low anoikis score subgroups (Fig. S7A). Subsequent TIDE (tumor immune dysfunction and exclusion) prediction results demonstrated that low anoikis score patients have more likelihood to respond to immune checkpoint blockade (Figs. S7B–E). The response to small molecular compounds was compared between the two subgroups. The anoikis score patients owned the more sensitivity to three CTRP-derived compounds, and nine PRISM-derived compounds (Figs. S7F–I).

The survival implication of the prognostic genes from the anoikis-related model was further assessed in TNBC. High expression of ALDH9A1, BEX2, CHADL, GLDC, GSTA1, NMNAT3, and SMPDL3B was connected to better prognosis, while high expression of CERCAM, COL5A3, COL18A1, EFNB2, HEYL, IGSF21, SERPINE1, and ZNF703 was linked with worse prognosis (Fig. S8). Gene set enrichment analysis result revealed that T cell receptor signaling pathway, cell cycle, cytokine receptor interaction, homologous recombination pathway, *etc*. were significantly enriched in the low expression group of the better prognostic genes (ALDH9A1, BEX2, CHADL, GSTA1, NMNAT3), while focal adhesion, ECM receptor interaction, chemokine signaling pathway, and cell adhesion molecules cams were significantly enriched in the high expression group of the most worse prognostic genes (CERCAM, COL5A3, COL18A1, HEYL, SERPINE1) (Table S6). We further evaluated their expression level using the TNBC paired samples from the TCGA database and found EFNB2, IGSF21, and ZNF703 showed a significant difference (Fig. S9). The normal breast epithelial cells MCF 10A as well as TNBC cell lines BT20, MDA-MB-231, and MDA-MB-468 were employed to further validate it and the result indicated that these genes were significantly reduced in TNBC (Fig. S10A, B). Out of these three genes, we selected the EFNB2 gene with the largest expression difference for further validation. By lentiviral infection, we constructed a stable transient cell line with enhanced EFNB2 expression in MDA-MB-231 cells. The successful construction of this cell line was confirmed through Western blot and quantitative reverse transcription PCR experiments (Fig. S11A, B). To investigate EFNB2's role in TNBC, we examined its impact on cell proliferation using a CCK-8 assay in MDA-MB-231 cells and found that overexpression of EFNB2 significantly inhibited cell proliferation (Fig. S11C). Furthermore, flow cytometry revealed a notable increase in early and late apoptosis after EFNB2 overexpression, indicating increased cell apoptosis (Fig. S11D). Finally, the transwell assay showed a significant reduction in cell invasion following EFNB2 overexpression (Fig. S11E). All of the above data demonstrated that EFNB2 was involved in regulating the biological process of TNBC, thereby validating our bioinformatics analysis.

Overall, these findings unveiled anoikis heterogeneity in TNBC and proposed the anoikis-based classification and scoring system. Each anoikis-based subtype or subgroup presented distinct and heterogeneous molecular and clinical traits, and treatment responses to the existing treatment modalities of TNBC, which may assist in comprehending anoikis and formulating an appropriate treatment choice directed towards the complete elimination of TNBC.

## Author contributions

KF conceived and designed the study. ZX and SJ conducted most of the experiments and data analysis, and wrote the manuscript. ML, CY participated in collecting data and helped to draft the manuscript. All authors contributed to the article and approved the submitted version.

## Conflict of interests

The authors declare that the research was conducted in the absence of any commercial or financial relationships that could be construed as a potential conflict of interests.

## Funding

The research was supported by a Ningxia Reproductive Disease Clinical Medical Research Center Project(2023LCYX003),Ningxia Hui Autonomous Region Natural Science Foundation Project (2022AAC03748), Ningxia Hui Autonomous Region Natural Science Foundation Project (2021AAC03523),Yinchuan Science and Technology Innovation Project (2023SF25). Basic research project of Yinchuan Maternal and Child Health Hospital (2022NYFYCX05),Basic research project of Yinchuan Maternal and Child Health Hospital (2023NYFYCX01).

## Data availability statement

The original contributions presented in the study are included in the article/Supplementary Material. Further inquiries can be directed to the corresponding author.

## Figures and Tables

**Figure S1:**
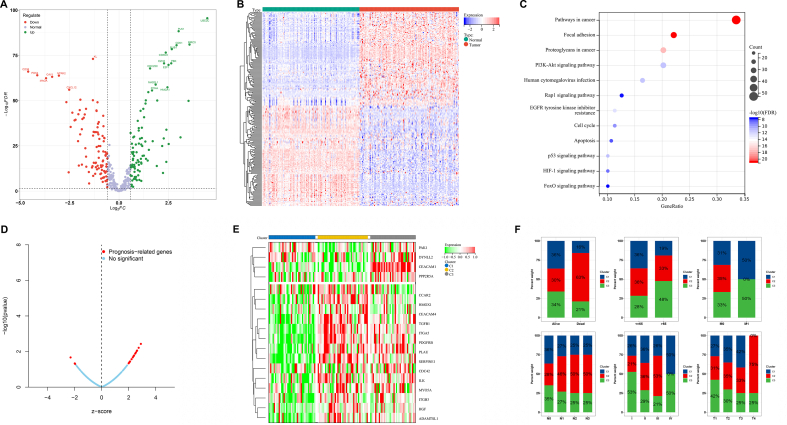
Characterization of anoikis-related genes and development of three anoikis-based subtypes with diverse molecular and clinical features across TCGA TNBC samples. **(A, B)** The volcano and heatmap diagrams illustrating the differential expression of anoikis genes in TNBC versus normal tissues. **(C)** The enrichment of KEGG pathways on the differentially expressed anoikis genes. **(D)** The volcano plots showing the prognostic differentially expressed anoikis genes (denoted as red dots). **(E)** The expression features of the prognostic differentially expressed anoikis genes across the three anoikis-based subtypes. **(F)** The distribution of clinical parameters in each subtype.

**Figure S2:**
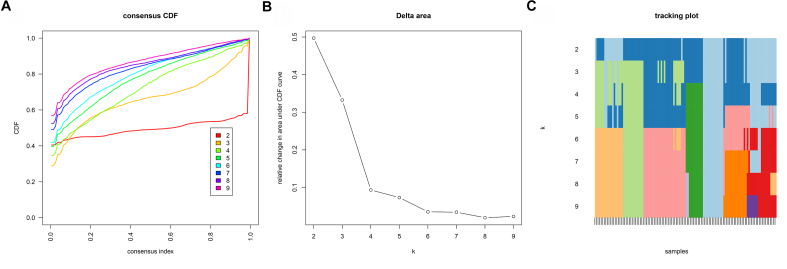
Consensus clustering analysis on TCGA TNBC samples in accordance with the prognostic differentially expressed anoikis genes. **(A)** Consensus CDF curves at k = 2–9. **(B)** Relative alteration in area under CDF curves. **(C)** Tracking plot of sample classification. CDF, cumulative distribution function.

**Figure S3:**
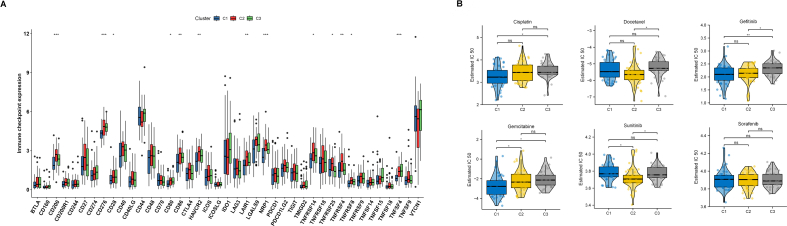
Three anoikis-based subtypes with immune checkpoints and drug sensitivity. **(A)** The expression of immune checkpoints in the three subtypes. **(B)** The estimated IC50 values of chemotherapy or targeted drugs in each subtype.

**Figure S4:**
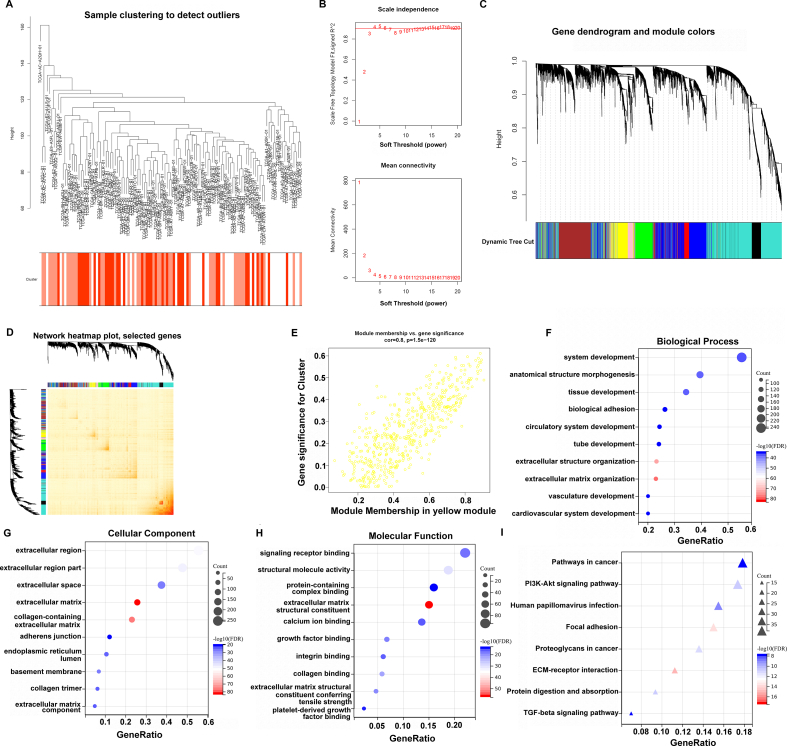
Selection of anoikis subtyping-related genes. **(A)** Sample dendrogram, and heatmap of anoikis-based subtyping. **(B)** Scale independence or mean connectivity under a gradient of soft threshold powers. **(C)** Gene dendrogram, and merged co-expression modules marked by unique colors. **(D)** The network heatmap plot illustrating the associations between the selected genes. The darker the yellow, the stronger the association. **(E)** The scatter plots displaying the associations of module membership of the yellow module with gene significance of anoikis subtyping. **(F–I)** The enrichment of biological processes, cellular components, molecular functions, and KEGG pathways on the yellow module genes.

**Figure S5:**
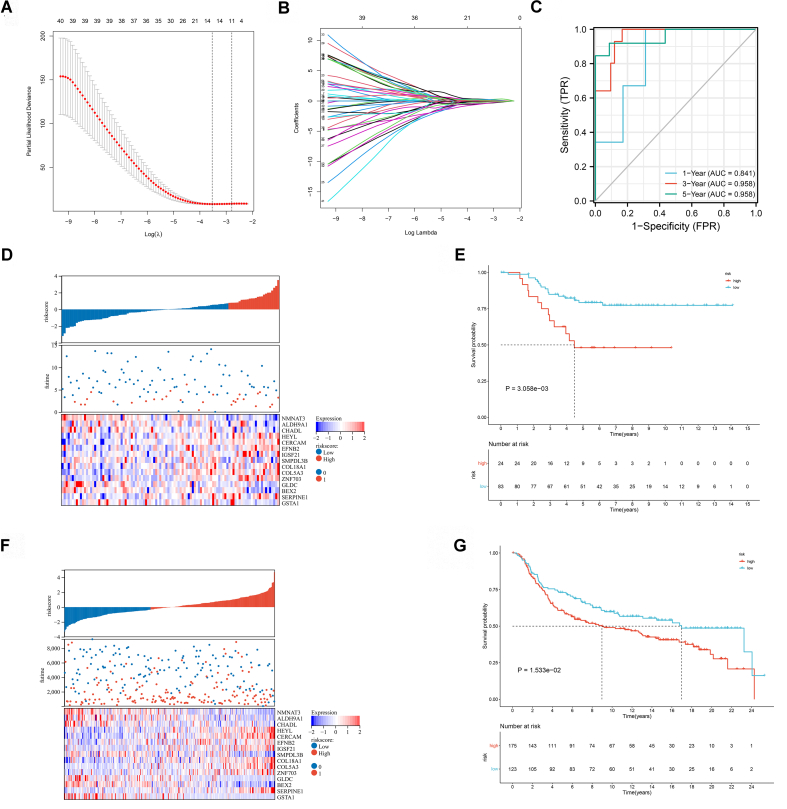
Definition of an anoikis scoring system for TNBC. **(A)** LASSO cross-validation for identifying the value of λ with the least error. **(B)** LASSO coefficient profiling. **(C)** Receiver operating characteristic (ROC) curves under the endpoints at one, three, and five years in the TCGA TNBC dataset. **(D–G)** Validation of distribution of anoikis score, survival time, and gene expression as well as survival probability of low or high anoikis score group in the (D, E) GSE58812 and (F, G) METABRIC datasets.

**Figure S6:**
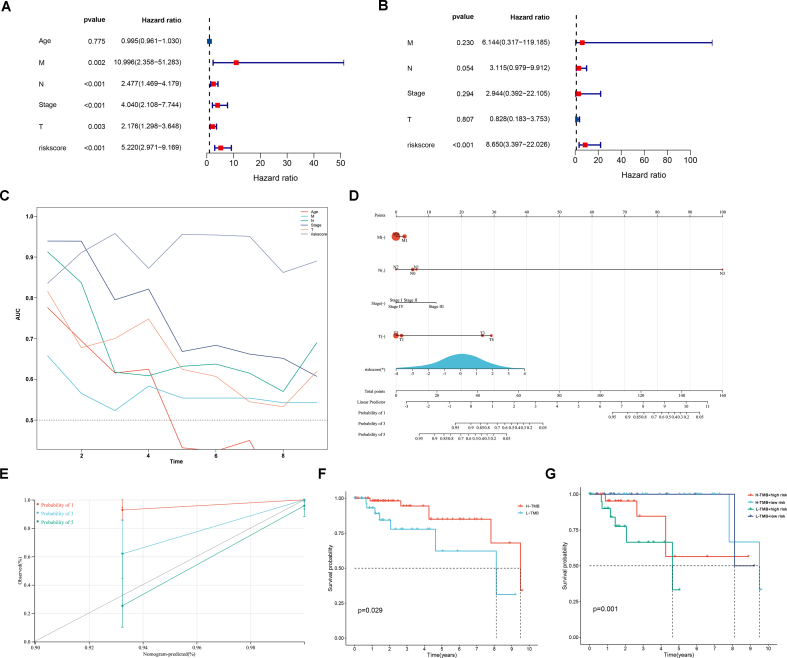
The anoikis score independently and reliably estimates patient prognosis in TCGA TNBC dataset. (A, B) Uni- and multivariate-cox regression analyses on the anoikis score, and clinical parameters with prognosis. (C) Time-independent ROC curves of the anoikis score, and clinical parameters. (D) The nomogram composed of prognostic variables for survival estimation. (E) Calibration curves demonstrating the predictive accuracy of the nomogram. (F) Survival analysis of the tumor mutation burdan (TMB). (G) Survival probability of the TMB and anoikis score subgroups.

**Figure S7:**
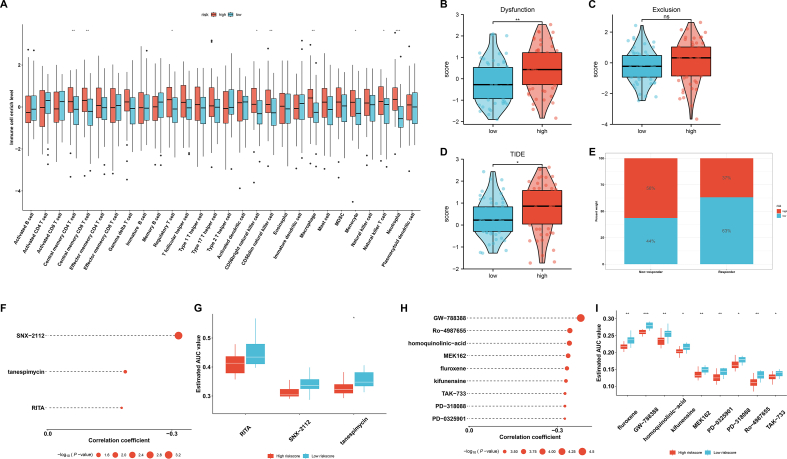
The anoikis score is associated with responses to ICB and small molecular drugs. **(A)** The infiltration of immune cells in low or high anoikis score samples. **(B–E)** Dysfunction, exclusion, and TIDE (tumor immune dysfunction and exclusion) scores, and predicted responders to ICB in the two subgroups. **(F, G)** The associations of anoikis score with AUC values of CTRP-derived compounds, and comparison of their AUC values between subgroups. **(H, I)** The associations of anoikis score with AUC values of PRISM-derived compounds, and comparison of their AUC values between subgroups. ICB, immune checkpoint blockade. AUC, area under curve.

**Figure S8:**
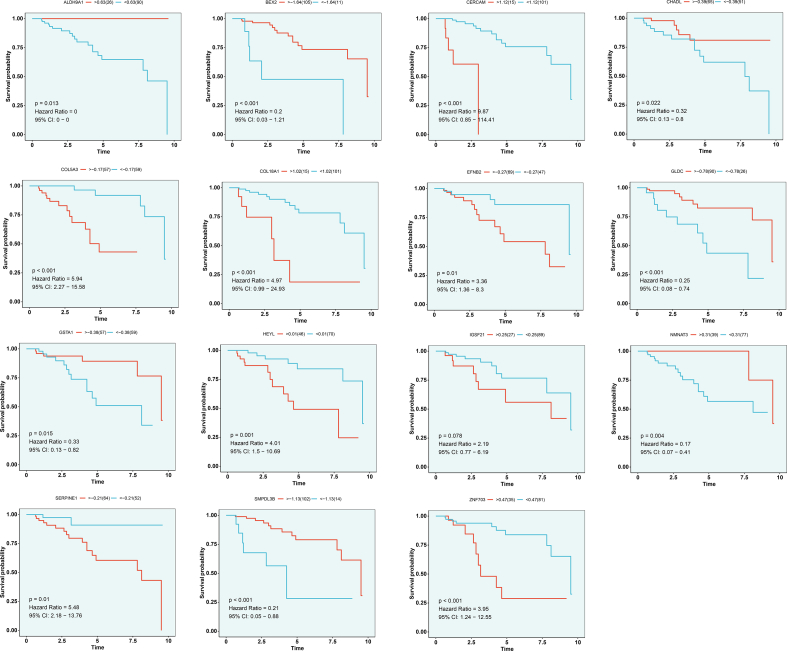
Survival probability of patients with low or high expression of ALDH9A1, BEX2, CERCAM, CHADL, COL5A3, COL18A1, EFNB2, GLDC, GSTA1, HEYL, IGSF21, NMNAT3, SERPINE1, SMPDL3B, and ZNF703.

**Figure S9:**
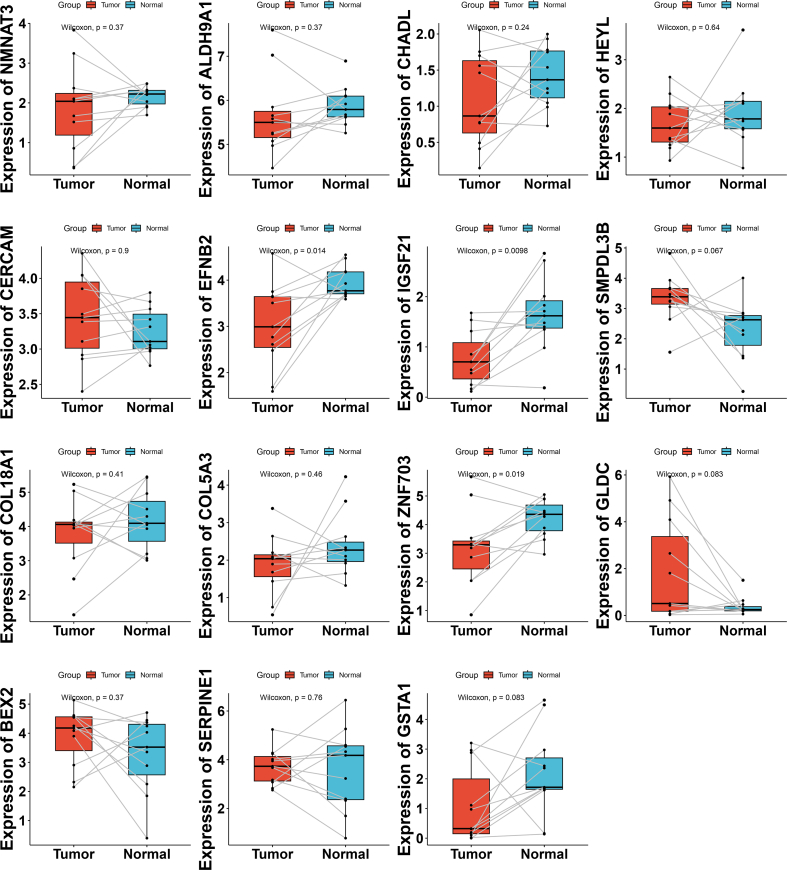
Evaluation of gene expression including NMNAT3, ALDH9A1, CHADL, HEYL, CERCAM, EFNB2, IGSF21, SMPDL3B, COL18A1, COL5A3, ZNF703, GLDC, BEX2, SERPINE1, and GSTA1in paired samples of TNBC.

**Figure S10:**
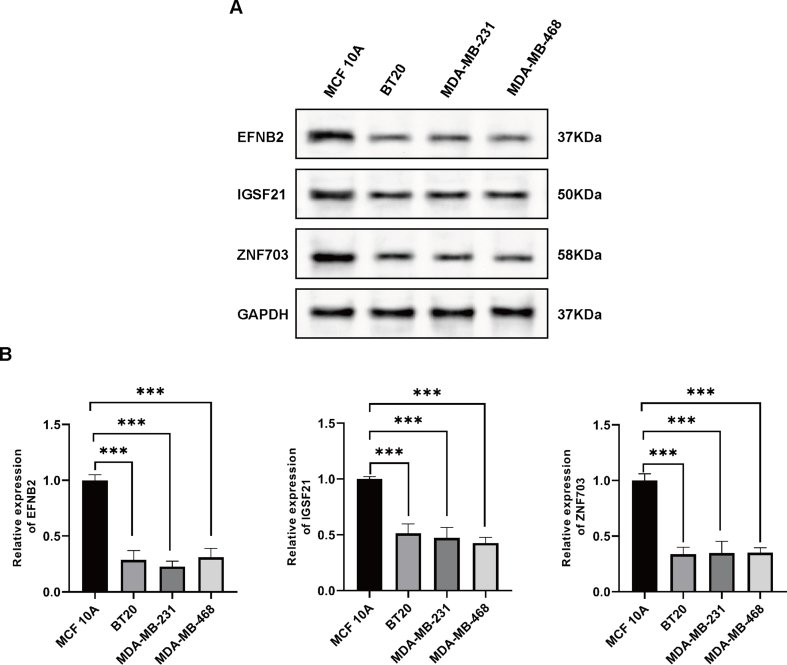
Expression of EFNB2, IGSF21, and ZNF703 genes in triple-negative breast cancer. **(A)** Western blot detection of gene expression in human normal breast epithelial cells MCF 10A and triple-negative breast cancer cells BT20, MDA-MB-231, and MDA-MB-468. **(B)** Quantitative reverse transcription PCR was performed to detect gene expression in human normal mammary epithelial cells MCF 10A and triple-negative breast cancer cells BT20, MDA-MB-231, and MDA-MB-468.

**Figure S11:**
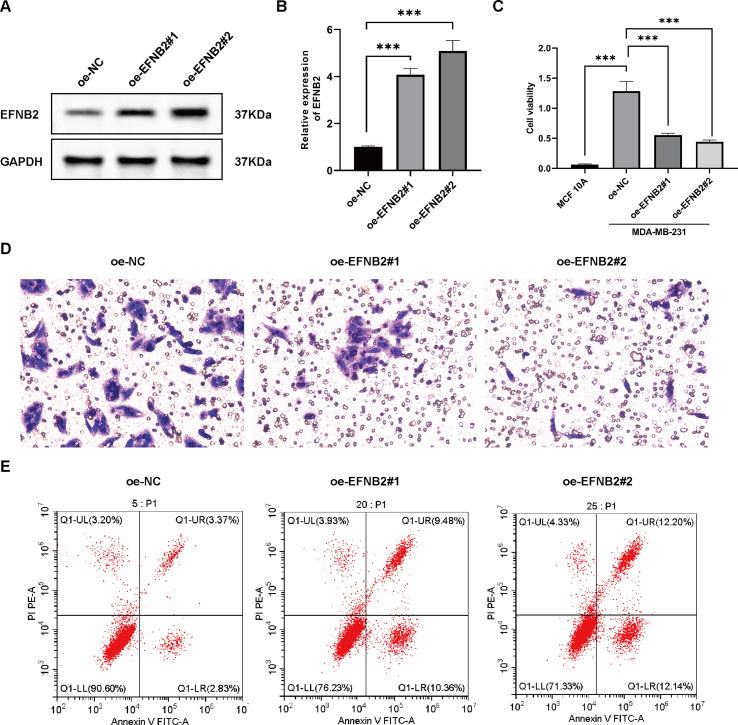
EFNB2 is implicated in the regulation of proliferation, apoptosis, and invasive function of triple-negative breast cancer cells. **(A, B)** Stable transient cell lines overexpressing EFNB2 were constructed in MDA-MB-231 cells. **(C)** CCK-8 assay was performed to detect the functional effects of overexpression of EFNB2 on the proliferation of MDA-MB-231 cells. **(D)** Transwell assay was used to investigate the functional effects of overexpression of EFNB2 on the invasive function of MDA-MB-231 cells. **(E)** Flow assay for the influence of overexpression of EFNB2 on apoptosis in MDA-MB-231 cells.
