# Supplementary material for: Heterogeneity of anoikis in triple-negative breast cancer subtyping and therapeutic implications
Source: Genes Dis. 2024 Mar 4;12(1):101255. doi: 10.1016/j.gendis.2024.101255 (PMC12053675; doi:10.1016/j.gendis.2024.101255)
Supplement: Multimedia component 1 [file mmc1.docx]

**Materials and methods:**

**Sample collection**

The list of 434 anoikis genes was queried from the GeneCard database (**Table S1**). Transcriptome data on 116 TNBC and 113 normal tissues were obtained from The Cancer Genome Atlas (TCGA). Additionally, clinical parameters of these TNBC cases were downloaded (**Table S2**). The GSE58812 (n=107) from the Gene Expression Omnibus ^1^, and METABRIC (n=298) datasets were utilized for external verification.

**Consensus clustering analysis**

Differential expression analysis on anoikis genes was conducted in TNBC and normal groups with the threshold of adjusted p<0.01 and |log2fold change|>0.585. The prognostic anoikis genes (p<0.05) were selected using the univariate cox regression analysis. Unsupervised clusters of TCGA-TNBC samples were analyzed via ConsensusClusterPlus package based upon their transcriptional values ^2^. Consensus matrix and cumulative distribution function (CDF) were conducted for ensuring the appropriate number of clusters. The clusters were proven via principal component analysis (PCA) through broom package. The gene expression features in each cluster were visualized via ComplexHeatmap package.

**Functional enrichment analysis**

Enrichment of Gene Ontology (GO) or Kyoto Encyclopedia of Gene and Genome (KEGG) pathways was analyzed via clusterProfiler package ^3^. The “h.all.v7.5.1.symbols” gene set was acquired from the Molecular Signatures Database ^4^. Through GSVA package, the activity of well-established hallmarks was estimated ^5^. TIDE computational approach was adopted for predicting immune checkpoint blockade (ICB) response in accordance with signatures of T cell dysfunction and exclusion ^6^.

**Assessment of tumor immunity**

By executing single sample gene set enrichment analysis (ssGSEA), the abundance of diverse immune cell populations was estimated. The expression values of well-established immune checkpoints were also computed.

**Weighted gene co-expression network analysis (WGCNA)**

WGCNA package was employed for co-expression analysis ^7^. An appropriate soft threshold value was selected for transforming the adjacency matrix to the topological overlap matrix. Pearson correlation analysis on co-expression modules and anoikis subtyping was conducted. The genes in the co-expression module with the strongest association with anoikis subtyping were selected as anoikis subtyping-related genes.

**Generation of an anoikis scoring system**

Least absolute shrinkage and selection operator (LASSO) regression analysis on prognostic anoikis subtyping-related genes with prognosis was executed via glmnet package. Next, ten-fold cross-validation was conducted, and the genes were eventually chosen based upon the point with the minor error. Anoikis score was calculated as the sum of the gene expression and given coefficients. With the median score, patients were classified as low or high anoikis score subgroup.

**Screening small molecule compounds**

Drug sensitivity profiles of human cancer cell lines were acquired from the Cancer Therapeutics Response Portal (CTRP) or the PRISM project, and the response to small molecule compounds were estimated ^8^.

**Cell Culture and Transfection**

Human normal mammary epithelial cells (MCF 10A) and triple-negative breast cancer cell lines BT20, MDA-MB-231, MDA-MB-468 were cultured in DMEM medium containing 10% FBS and 1% penicillin-streptomycin solution. Cells were grown in a cell culture incubator at 37°C and 5% CO_2_. Cells were passaged when they were in the logarithmic growth phase, and the passaged cells were used for subsequent experimental validation. Construction of recombinant lentiviral vector overexpressing EFNB2 and empty vector. MDA-MB-231 cells were cultured in lentiviral medium containing polybrene (10 μg/mL) for 48 hours, and then the viral medium was replaced with DMEM medium containing 10% fetal bovine serum. After 72 hours of transfection, cells were screened with 2 μg/mL puromycin to establish a cell line stably overexpressing EFNB2.

**CCK-8**

Cells were inoculated in 96-well plates at a density of 2 × 10^4^ cells per well with 100 μL of DMEM medium containing 10% fetal bovine serum. 18-24 h later, the supernatant was removed, and the cells were placed in DMEM medium containing 10 μL of CCK-8 solution and incubated for 3 h at 37°C with 5% CO_2_. The optical density was measured at 450 nm.

**Western Blot**

The appropriate amount of cells was collected, and each group of cells was lysed with RIPA buffer(P0013B, Beyotime), and the protein concentration was determined by BCA method(P0009, Beyotime). 25 μg of total protein was added to each well for protein SDS-PAGE electrophoresis, and then transferred to a PVDF membrane. The membranes were incubated with 5% skimmed milk powder for 1h, washed three times with 1×TBST solution, and then incubated with EFNB2 (sc-398735, Santa Cruz, 1:500), IGSF21 (21465-1-AP, Proteintech, 1:1000), ZNF703 (21075-1-AP, Proteintech, 1:1000), and GAPDH (60004-1-Ig, Proteintech, 1:50000) primary antibodies overnight at 4°C. After washing the membrane with 1×TBST, the secondary antibody was added and incubated on a shaker at 37°C for 2 h. The membrane was washed three times with 1×TBST solution, and the protein expression was detected by chemiluminescence imager.

**qRT-PCR**

Total RNA was extracted using Trizol reagent(19201ES60, YEASEN) and placed in 1.5 ml EP tubes for 5 minutes. Then add 200 μL of chloroform and centrifuge for 15 minutes at 4°C, 12,000 rpm. The upper aqueous phase was transferred and mixed with 400 μL isopropanol. After centrifugation, the supernatant was discarded and the precipitate was dissolved in 20 μL of DEPC water(10601ES60, YEASEN). cDNA was synthesized by reverse transcription under the conditions of the Reverse Transcription Reagent(11123ES60, YEASEN). cDNA was diluted 10-fold and amplified by real-time fluorescence quantitative PCR according to the reaction system. Primer sequences for qPCR synthesis are listed in Table S5.

**Table S5. Primer Sequences**

| **Gene** | **Forward Primer Sequence(5**'-**3**') | **Reverse Primer Sequence(5**'-**3**') |
| --- | --- | --- |
| GAPDH | TGAAGGTCGGAGTCAACGGATTTGG | TGATGGCATGGACTGTGGTCATGAG |
| EFNB2 | CCTTTGTAAAACCAAATCCAGGTTC | GTGCTTCCTGTGTCTCCTCC |
| IGSF21 | GCGTCTGCCTGCTGCTC | CTCGGGCAGCCTCACAG |
| ZNF703 | TCAGCCCCATTGAGCTGGAC | GAAGCTGGACTTGTCCTCGG |

**Transwell**

Using a Transwell cell chamber with a pore size of 8 μm, 60 μL of Matrigel (1:8) was added to the upper cell chamber. The cells were then incubated at 37°C for 30 min under 5% CO_2_, digested with trypsin and resuspended in serum-free DMEM medium and diluted to a concentration of 1×10^5^ cells/mL. 200 μL of cell suspension was added to the upper chamber of the Transwell, while the lower chamber was inoculated with the same number of tumor cells and injected with complete medium (20% FBS) for 24 h. The upper chamber was then removed and fixed with methanol for 15 min at room temperature. Then the upper chamber was taken out, fixed with methanol for 15 minutes at 25℃, stained with crystal violet for 15-20 minutes and rinsed with 1×PBS for 3 times, and the field of view was randomly selected and photographed under the microscope.

**Flow cytometry to detect apoptosis**

The V-FITC/PI Apoptosis Detection Kit (ThermoFisher Scientific, USA) was used. First, cells were rinsed with pre-cooled PBS and then collected at a density of 5 × 10^5^ cells. Then, 1×binding buffer was prepared by diluting 5×binding buffer with ddH_2_O, added to each tube and incubated for 5 min under light protection. Finally, the results were analyzed using FlowJo software after detection by flow cytometry.

**Statistical analysis**

All the analyses were implemented utilizing R software (version 4.2.1). Continuous variables between two groups were compared via student’s t or Wilcoxon rank-sum test, with one-way analysis of variance or Kruskal-Wallis test for comparison between ≥3 groups. Categorical data were assessed via chi square test. Correlation analysis was achieved via Pearson or Spearman test. Kaplan–Meier survival curves were drawn and evaluated utilizing survival and survminer packages. Time-independent receiver operating characteristic (ROC) curves were plotted via timeROC package. Uni- and multivariate-cox regression analyses were implemented on variables with prognosis. A nomogram was built for estimating the survival probability utilizing rms package, and the predicted accuracy was appraised through calibration curves. Experimental data were analyzed using GraphPad Prism 8.0. and SPSS 20.0 software. Results are expressed as mean ± standard deviation. Differences between groups were analyzed by analysis of variance (ANOVA), and differences between two groups were analyzed by t-test. Statistical significance was set at p<0.05.

1. Jézéquel, P.; Loussouarn, D.; Guérin-Charbonnel, C.; Campion, L.; Vanier, A.; Gouraud, W.; Lasla, H.; Guette, C.; Valo, I.; Verrièle, V.; Campone, M., Gene-expression molecular subtyping of triple-negative breast cancer tumours: Importance of immune response. *Breast cancer research : BCR* **2015,** *17*, 43.

2. Wilkerson, M. D.; Hayes, D. N., ConsensusClusterPlus: a class discovery tool with confidence assessments and item tracking. *Bioinformatics* **2010,** *26* (12), 1572-3.

3. Yu, G.; Wang, L. G.; Han, Y.; He, Q. Y., clusterProfiler: an R package for comparing biological themes among gene clusters. *Omics* **2012,** *16* (5), 284-7.

4. Liberzon, A.; Birger, C.; Thorvaldsdóttir, H.; Ghandi, M.; Mesirov, J. P.; Tamayo, P., The Molecular Signatures Database (MSigDB) hallmark gene set collection. *Cell Syst* **2015,** *1* (6), 417-425.

5. Hänzelmann, S.; Castelo, R.; Guinney, J., GSVA: gene set variation analysis for microarray and RNA-seq data. *BMC Bioinformatics* **2013,** *14*, 7.

6. Jiang, P.; Gu, S.; Pan, D.; Fu, J.; Sahu, A.; Hu, X.; Li, Z.; Traugh, N.; Bu, X.; Li, B.; Liu, J.; Freeman, G. J.; Brown, M. A.; Wucherpfennig, K. W.; Liu, X. S., Signatures of T cell dysfunction and exclusion predict cancer immunotherapy response. *Nat Med* **2018,** *24* (10), 1550-1558.

7. Langfelder, P.; Horvath, S., WGCNA: an R package for weighted correlation network analysis. *BMC Bioinformatics* **2008,** *9*, 559.

8. Ghandi, M.; Huang, F. W.; Jané-Valbuena, J.; Kryukov, G. V.; Lo, C. C.; McDonald, E. R., 3rd; Barretina, J.; Gelfand, E. T.; Bielski, C. M.; Li, H.; Hu, K.; Andreev-Drakhlin, A. Y.; Kim, J.; Hess, J. M.; Haas, B. J.; Aguet, F.; Weir, B. A.; Rothberg, M. V.; Paolella, B. R.; Lawrence, M. S.; Akbani, R.; Lu, Y.; Tiv, H. L.; Gokhale, P. C.; de Weck, A.; Mansour, A. A.; Oh, C.; Shih, J.; Hadi, K.; Rosen, Y.; Bistline, J.; Venkatesan, K.; Reddy, A.; Sonkin, D.; Liu, M.; Lehar, J.; Korn, J. M.; Porter, D. A.; Jones, M. D.; Golji, J.; Caponigro, G.; Taylor, J. E.; Dunning, C. M.; Creech, A. L.; Warren, A. C.; McFarland, J. M.; Zamanighomi, M.; Kauffmann, A.; Stransky, N.; Imielinski, M.; Maruvka, Y. E.; Cherniack, A. D.; Tsherniak, A.; Vazquez, F.; Jaffe, J. D.; Lane, A. A.; Weinstock, D. M.; Johannessen, C. M.; Morrissey, M. P.; Stegmeier, F.; Schlegel, R.; Hahn, W. C.; Getz, G.; Mills, G. B.; Boehm, J. S.; Golub, T. R.; Garraway, L. A.; Sellers, W. R., Next-generation characterization of the Cancer Cell Line Encyclopedia. *Nature* **2019,** *569* (7757), 503-508.
